# A Comprehensive Analysis of Metabolomics and Transcriptomics Reveals Novel Biomarkers and Mechanistic Insights on Lorlatinib Crosses the Blood-Brain Barrier

**DOI:** 10.3389/fphar.2021.722627

**Published:** 2021-08-23

**Authors:** Wei Chen, Chunyu Li, Yafei Shi, Yujun Zhang, Dujia Jin, Mingyu Zhang, Mingming Bo, Guohui Li

**Affiliations:** ^1^ National Cancer Center, National Clinical Research Center for Cancer, Cancer Hospital, Chinese Academy of Medical Sciences and Peking Union Medical College, Beijing, China; ^2^ Institute of Materia Medica, Chinese Academy of Medical Sciences and Peking Union Medical College, Beijing, China

**Keywords:** lorlatinib, metabolomics, transcriptomics, artificial neural network, non-small cell lung cancer

## Abstract

Of late, lorlatinib has played an increasingly pivotal role in the treatment of brain metastasis from non-small cell lung cancer. However, its pharmacokinetics in the brain and the mechanism of entry are still controversial. The purpose of this study was to explore the mechanisms of brain penetration by lorlatinib and identify potential biomarkers for the prediction of lorlatinib concentration in the brain. Detection of lorlatinib in lorlatinib-administered mice and control mice was performed using liquid chromatography and mass spectrometry. Metabolomics and transcriptomics were combined to investigate the pathway and relationships between metabolites and genes. Multilayer perceptron was applied to construct an artificial neural network model for prediction of the distribution of lorlatinib in the brain. Nine biomarkers related to lorlatinib concentration in the brain were identified. A metabolite-reaction-enzyme-gene interaction network was built to reveal the mechanism of lorlatinib. A multilayer perceptron model based on the identified biomarkers provides a prediction accuracy rate of greater than 85%. The identified biomarkers and the neural network constructed with these metabolites will be valuable for predicting the concentration of drugs in the brain. The model provides a lorlatinib to treat tumor brain metastases in the clinic.

## Introduction

Lung cancer is the leading cause of cancer death in china and worldwide, and was responsible for an estimated 1.76 million deaths in 2018 (World Health Organization, 2018; Cao and Chen, 2019). Non-small cell lung cancer (NSCLC) accounts for up to 85% of all lung cancers (Majem et al., 2019). Brain metastasis, as a known complication of NSCLC, arises in about 10% of patients at the initial diagnosis of NSCLC and in approximately 30% patients with advanced NSCLC adenocarcinoma (Barnholtz-Sloan et al., 2004; Singh et al., 2020). For patients with anaplastic lymphoma kinase (ALK)-positive NSCLC, this frequent complication occurs in roughly 30% of patients even at the time of initial diagnosis, and in about 60% of patients over the course of first-line therapy (Guérin et al., 2015; Johung et al., 2016). As a consequence of the impermeability of the blood-brain barrier to many drugs in addition to effective systemic therapy, CNS metastases are emerging as a sanctuary site for tumor cell growth. Metastasis to the CNS can lead to poor prognosis and result in shortening of overall survival. At the same time, progressive deterioration of neurological and cognitive functioning caused by brain metastasis will also reduce the patient’s quality of life (Wanleenuwat and Iwanowski, 2020).

Anaplastic lymphoma kinase inhibitors are commonly employed for an oncogene-driven subset of NSCLC patients, targeting ALK rearrangement specifically, which produces in turn leads to generation of the ALK protein before causing tumor cells to grow and spread (Straughan D et al., 2016). Currently, the first generation ALK TKI (crizotinib) and the second-generation ALK TKIs (alectinib, brigatinib, ceritinib) are both recommended after updates to the NCCN Clinical Practice Guidelines in Oncology (NCCN Guidelines®) and NCCN Drugs and Biologics Compendium (NCCN Compendium®) for Non-Small Cell Lung Cancer for the treatment of ALK + NSCLC patients (Pinto et al., 2020; National Comprehensive Cacncer Network and NCCN eBulletin Newsletter). However, first generation ALK TKIs are not ideal for controlling the progression of central nervous system metastasis (Costa et al., 2015). Although the blood-brain barrier penetration of the second-generation ALK TKI has been enhanced compared with the first-generation ALK TKI, there is still an intense demand to improve control of CNS metastasis in NSCLC.

Lorlatinib, a third-generation inhibitor of anaplastic lymphoma kinase (ALK), can achieve higher exposures in the CNS when compared with previous generations of inhibitors (Shaw et al., 2017; Nagasaka et al., 2020). Due to high CNS permeability, which had been confirmed by PET imaging (Collier et al., 2017a; Collier et al., 2017b), lorlatinib possesses an impressive confirmed intracranial objective response rate ranging from 41.7 to 87.0% in ALK-positive patients with CNS metastasis (Shaw et al., 2017; Solomon et al., 2018; Shaw et al., 2019). Lorlatinib has an active role in the treatment and prevention of CNS metastasis in ALK-positive NSCLC patients (Bauer et al., 2020). In addition to the possible mechanism of minimizing p-glycoprotein-mediated efflux of relatively large (>400 Da) hydrophobic drugs (Schinkel, 1999; Seelig, 2020), our previous research showed that downregulating SPP1 and inhibiting VEGF, TGF-β may also be potential mechanisms for lorlatinib’s characteristics of effective brain penetration (Chen et al., 2020).

To further clarify the mechanisms of brain penetration by lorlatinib, ultra-performance liquid chromatography and quadrupole/time-of-flight mass spectrometry (UPLC-Q/TOF-MS) was applied for investigation of the dynamic changes in serum metabolites in mice in physiological conditions and after treatment with lorlatinib. Furthermore, potential biomarkers for prediction of lorlatinib concentration in the brain were identified.

## Materials and Methods

### Chemicals and Reagents

Lorlatinib (>99.9%) was obtained from MedChem Express (United States). Methanol, HPLC-grade, was purchased obtained from Fisher Chemicals (Pittsburgh, PA, United States). Acetonitrile, HPLC-grade, was obtained from Merck (Darmstadt, Germany). Purified water was produced by Millipore’s ultrapure water system (Millipore, Bedford, MA, United States). All other chemicals and reagents were of analytical grade unless otherwise indicated.

### Animals

All the animal-related experiments were conducted in accordance with guidelines of Institutional Experimental Animal Ethical Committee. SPF grade KM and ICR mice (weight: 18–20 g, age: 8 weeks) were obtained from the Beijing HFK Bioscience Co., Ltd. (License No. 11401300092657). All mice were given free access to normal diet and water during the experiment with an exception that mice were fasted for 12 h prior to drug administration. The experiment was conducted under standard breeding conditions with a temperature regime of 26°C day/18°C night, a relative humidity level of between 50 and 70 percent and a 12-h light/12-h dark photocycle. Mice weighing more than 21 g or less than 18 g were excluded from the analysis. Additionally, mice that suffered accidental injury and/or bleeding during the study were excluded from the analysis and finally, mice that died unexpectedly during the study were excluded from the analysis.

### Experimental Design for Metabolomics

After 3 days of acclimatization, KM mice (weight: 18–20 g, age: 8 weeks) acquired for this study were weighed and randomly distributed into 2 groups: a lorlatinib group and a non-lorlatinib group. The mice in the non-lorlatinib group were orally administrated with physiological saline solution and the mice in the lorlatinib group were orally administered with 10 mg/kg lorlatinib (the concentration of lorlatinib solution: 1 mg/ml). Blood was collected from mice in both groups at 0.5, 1, 2, 4, 8, and 24 h after administration. Serum was exacted from the collected blood and stored at −80°C for further pretreatment and analysis.

### Sample Collection

Blood samples were collected from each mouse via orbital sinus at 0.5, 1, 2, 4, 8, and 24 h after lorlatinib administration and transferred to a non-heparinized tube. The blood was allowed to clot at room temperature before being centrifuged to separate serum, which was then stored at −80°C until further sample preparation.

### Sample Handling for Metabolomics

Methanol (150 μL) with an internal standard, 2-chlorophenylalanine (20 mg/ml), was added to 50 μL serum samples in 1.5 ml centrifuge tubes followed by vortexing for more than 30 s. The mixture was centrifuged at 14,000 rpm for 10 min at 4°C. 120 μL of supernatant was collected from the centrifuged mixture and spin-dried in a centrifuge tube. Sixty μL of 75% methanol was used to re-dissolve the sample, which was then centrifugated at 12,000 rpm for 10 min to separate 15 μL of supernatant as the final sample that was analysed using mass spectrometry.

### Lorlatinib Concentration Analysis

We have previously developed a rapid liquid chromatography-tandem mass spectrometry (LC-MS/MS) method for analysis of the concentration of lorlatinib in mouse serum (Chen et al., 2019). Methanol was used to precipitate protein in samples and lorlatinib was separated on a C18 column by gradient elution (0.1% of formic acid and methanol) and detected in the positive-ion mode with m/z 407.28 [M + H]^+^.

### Liquid Chromatography and Mass Spectrometry Conditions

Waters Xevo G2-XS QTOF/MS (Waters, Manchester, United Kingdom) was utilised for chromatographic analysis. A reverse phase column, UPLC HSS T3 C18 (100 mm × 2.1 mm, 1.8 μm), was used for chromatographic separation with the column temperature set to 40°C. The detection wavelength was set at 275 nm. The optimal mobile phase consisted of ultrapure water with 0.1% formic acid as solvent A and acetonitrile with 0.1% formic acid as solvent B. The gradient conditions were as follows: 0–1 min, 95 to 95% A; 1–9 min, 95 to 60% A; 9–19 min, 60 to 10% A; 19–21 min, 10 to 0% A; 21–25 min, 100 to 100% B. The sample injection volume was 4 µL. To verify the accuracy and reproducibility, the sample run sequence was randomized and quality control (QC) samples were prepared and analyzed every 10 samples. All samples were maintained at 4°C during the experimental period.

For mass spectrometry profiling, Waters Xevo G2-XS QTOF/MS equipped with an electrospray ionization sources (ESI) (Waters Corporation, Manchester, United Kingdom), in which both positive and negative ESI was produced and detected. All mass scans were acquired under MSE mode (specifically, ESI Continuum mode). Mass detection was operated using the following setting parameters: drying gas (N2); flow rate, 800 L/h; gas temperature, 350°C; capillary voltage, 2.2 kV (ESI-) and 2.5 kV (ESI+); skimmer, 40 V; collision energy, 10–40 EV. Leucine enkephalin (m/z 556.2771 in ES+ and 554.2615 in ES-) was used as the external standard substance to perform online mass calibration for all the detection runs. Masslynk 4.1 software was used to collect data, with detected molecular weights ranging from 50 to 1,200 Da.

### Data Processing of Metabolomics

The mass data were preprocessed by MetaboAnalyst 3.0 (www.metaboanalyst.ca/) to generate a normalized data matrix. For multivariate analysis, the data matrix was introduced into SIMCA-P 14.1 software (Umetrics, Umea, Sweden). Unsupervised Principal Component Analysis (PCA) was employed to describe and identify the differences and relationships between samples. The supervised Orthogonal Partial Least Squares Discriminant Analysis (OPLS-DA) model was constructed to mine for different metabolites. S-plots were created to confirm the result, as such, to avoid false positives: according to whether the variables were distributed in the neutral position, it could be determined whether there were significant alterations. OPLS-VIP parameter was applied to the metabolomics profiles of the experimental animal groups to achieve an improved certainty of the variables with the most significant contribution. Variables representing metabolites with a vip of more than 1, if the |p (corr)| ≥ 0.5, *p* value < 0.05 and folder change>2 or<0.5 at the same time, were considered as potential biomarkers. Molecules representing the potential biomarkers were identified by the online Human Metabolome database (https://hmdb.ca/) search engines based on the accurate mass data. The list of compound labels was uploaded to MetaboAnalyst 5.0 (http://www.metaboanalyst.ca/) and the pathway enrichment analyses were performed by the Pathway Analysis module to identify the most relevant pathways involved in the conditions of the study.

### RNA-Seq and Data Analysis

In our previous study, SD rats were randomly divided into groups (Chen et al., 2020). After cardiac perfusion with saline, the brain tissue of rats in the control group and in the lorlatinib administration group were taken for sequencing, which was completed at the BGI-Shenzhen. The library preparation included the following steps: mRNA isolation, RNA fragmentation, cDNA strand synthesis, ends reparation, A-tailing, adapter ligation, linker addition, PCR reaction and purification of products. The data obtained from sequencing, namely raw reads, was subjected to quality control (QC) to determine whether the sequencing data was suitable for subsequent analysis. After passing the quality control, the filtered clean reads were compared to the reference genome. On this basis, according to the statistical comparison rate and the distribution of reads on the reference sequence, it was judged whether the comparison result passed the second quality control (QC of alignment). Finally, gene quantitative analysis and various analyses of gene expression levels were carried out, these included: principal component, correlation, differential gene screening, etc., and the differentially expressed genes were subjected to GO function enrichment analysis and pathway enrichment analysis.

### Preliminary Verification of Expression Levels of Key Proteins at the Blood-Brain Barrier of Mice After Lorlatinib Administration

ICR mice were randomly divided into three groups, with 3 mice in each group. The animals were fasted overnight without water before administration, and lorlatinib was given at a dose of 10 mg/kg (0.1 ml/10 g) after fasting. Brain tissue samples were taken at 30 min, 2 h, and 4 h after administration. Finally, the expression levels of OPN and related key proteins was detected by Western blotting.

### Western Blotting

The brain tissue of mice was lysed with lysis buffer (Solarbio, whole protein extraction kit, cat. no. BC3710) to extract total protein. The protein concentration was determined by using a BCA assay (NCM biotech, BCA protein assay kit, cat. no. WB6501). After treatment with protein loading buffer, a 10% PAGE precast gel was used for protein electrophoresis. Subsequently, the protein was transferred to a PVDF membrane (Millipore, IPVH00010). After blocking with 7% fat-free milk at room temperature for 1 h, the membrane was incubated with primary antibody overnight at 4°C. The primary antibodies used in this study were OPN (1:1,000, abcam, cat. no. ab8448) and β-actin (1:1,000, Bioss, cat. no. bs-0061R). After washing 5 times with TBST containing 0.01% Tween-20 at room temperature (3 times for 5 min, 2 times for 10 min), the blot was incubated with goat anti-rabbit IgG horseradish peroxidase (1:10,000, ZSGB-BIO, cat. no. ZB-2301) at room temperature for 1 h. After washing again with TBST, the immunoblots was visualized with a chemiluminescence reagent (APPLYGEN, Super ECL Plus, cat. no. P1050), and the gray value of the immunoblots was semi-quantified using ImageJ software.

### Artificial Neural Network Model for Prediction

In previous studies, the blood and brain concentrations of lorlatinib in 48 mice were measured by Liquid Chromatography-Mass Spectrometry after administration (Chen et al., 2019). Based on this data, we further calculated the drug brain/blood distribution coefficient for each mouse. After determining the median, the mice were divided into high-coefficient level and low-coefficient level groups based on the comparison of cerebral blood distribution coefficient and the calculated median; these groups were represented by 1 and 0 respectively. Taking the abundance of metabolic markers as the independent variable, a neural network was constructed to predict the size of the blood-brain distribution coefficient. 70% of the data was selected randomly to be part of the training set and the remaining 30% data was used in the test data set.

## Result

### Metabolomics Analysis of Serum

**FIGURE 1:**
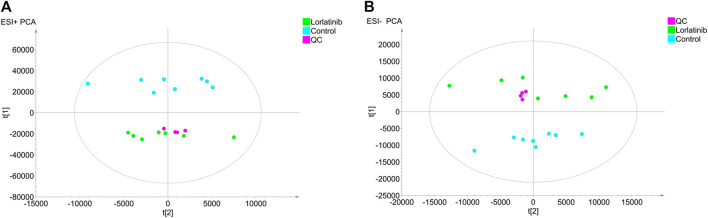
Principal component analysis (PCA) score plots obtained from the lorlatinib and control groups in positive **(A)** and negative **(B)** electrospray ionization source (ESI) mode.

The untargeted mass data collected by LC-IT-TOF/MS in positive and negative ion modes were analyzed using PCA to investigate the differences between the principal components of the control group and the lorlatinib group. PCA score scatter plots were illustrated in Figure 1A (ESI + mode) and Figure 1B (ESI- mode). The tightly grouped distribution characteristics of the quality control samples shown in both two figures indicated that the instrument was stable throughout the analytical process. Data generated on analysis of serum samples from the control group and the lorlatinib group gathered in distinct areas of the PCA score scatter plots, indicating substantial differences at the metabolite level between two groups.

**FIGURE 2:**
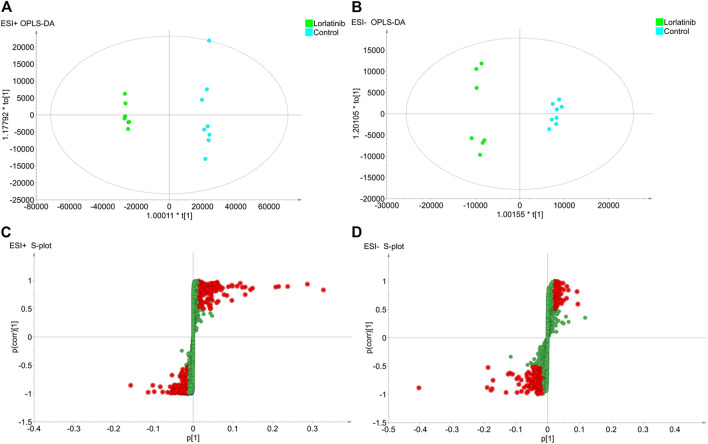
The results of OPLS-DA modelling using the data from the lorlatinib and non-lorlatinib groups in positive **(A)** and negative **(B)** electrospray ionization source (ESI) mode and the S-score plots constructed from the results in positive **(C)** and negative **(D)** mode.

To further investigate the potential differential metabolites between the two groups, the supervised Orthogonal Partial Least Squares Discriminant Analysis (OPLS-DA) model was established in order to identify the relationship between metabolite expression level and sample group and to make predictions regarding the sample category. As shown in the OPLS-DA scores plot for data generated in the ESI + mode (Figure 2A) and the ESI- mode (Figure 2B), the two sample groups clustered in different areas of the figure, indicating that the model could predict the classification of the two samples groups. The evaluation parameters R^2Y and Q2 of the OPLS-DA model were 0.997 and 0.984, respectively, in the ESI + mode and 0.989 and 0.935, respectively, in the ESI- mode. With the R^2Y and Q2 being greater than 0.5, this suggested that not only did the model have a satisfactory interpretation rate of the matrices, but also that the model could fit and predict accurately.

An S-plot (Figure 2C and Figure 2D), as an implement for visualization and interpretation of OPLS discriminate analysis, was carried out to identify statistically significant metabolites based on their reliability and contributions to the model. The variables appearing at the top or bottom of the S-plot had a significant contribution to modeled class designation, while those appearing in the middle were considered to contribute less. Variables were classified according to their explanatory power. Predictors with a VIP of larger than 1 were the most relevant for explaining classification and were marked in red in the S-plot if, at the same time, the absolute values of their p (corr) were greater than or equal to 0.5.

**FIGURE 3:**
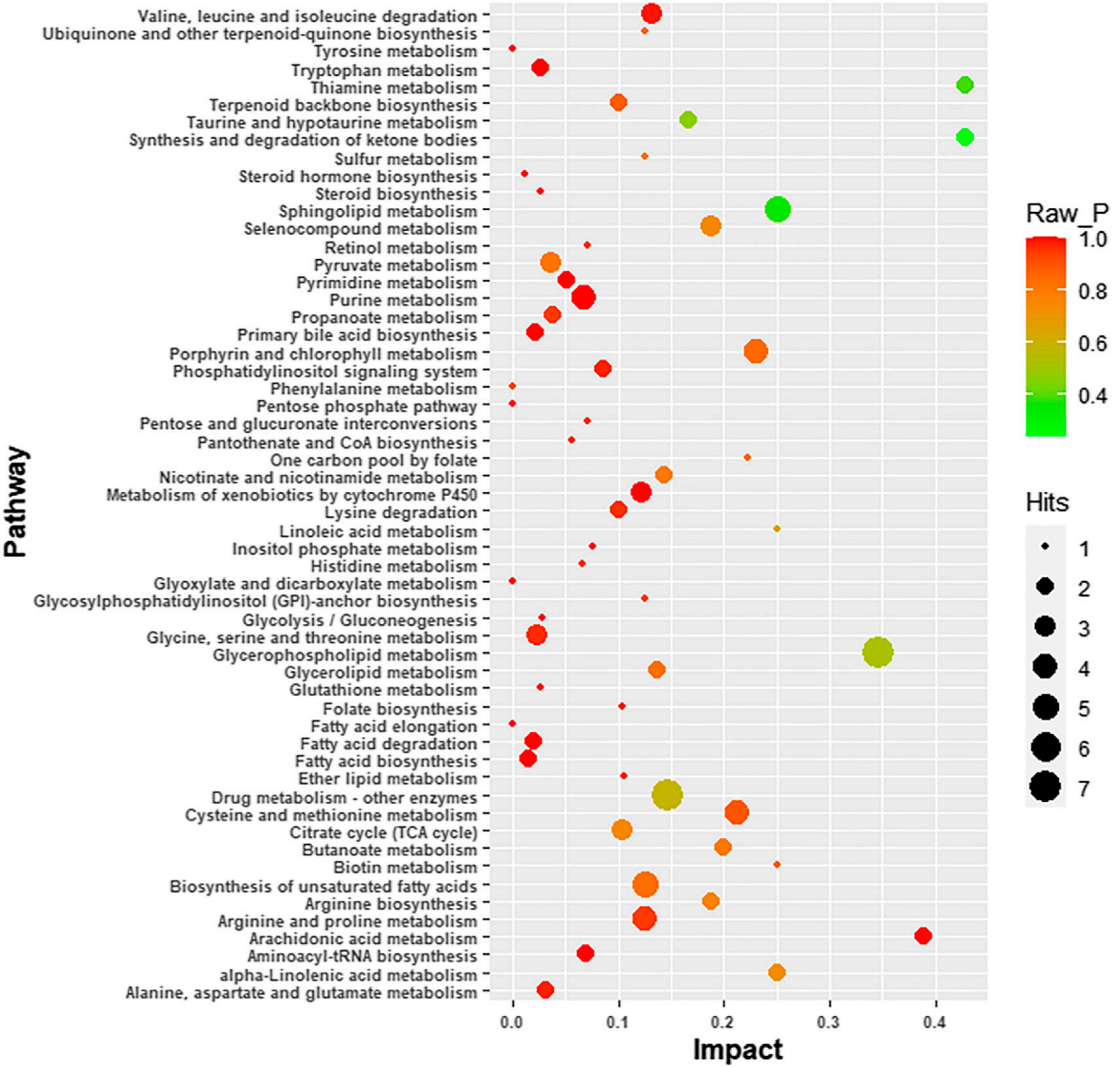
Schematic diagram of the altered metabolic pathways.

**FIGURE 4:**
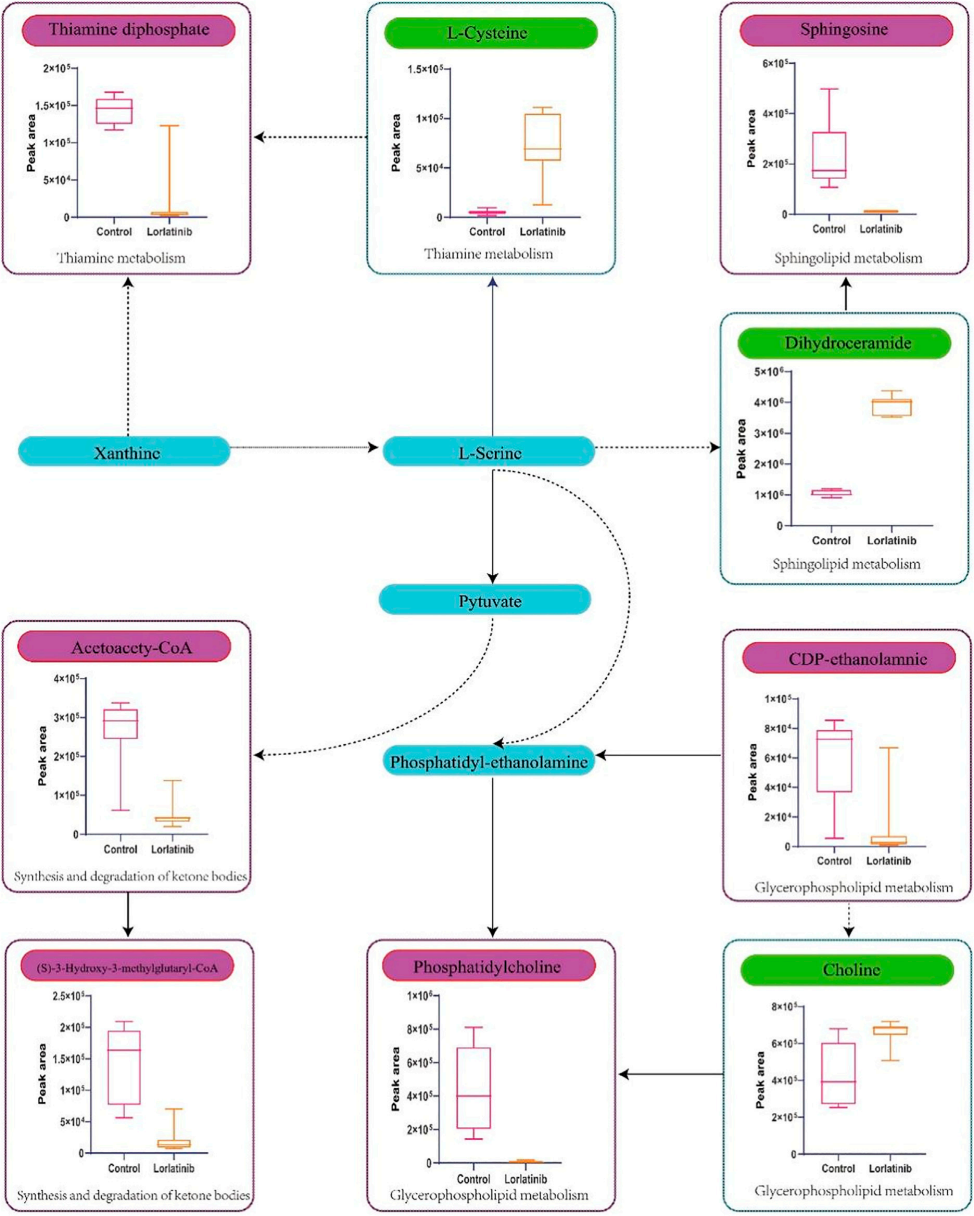
Schematic diagram of the metabolic pathways related to lorlatinib and the trends of biomarkers enriched in these metabolic pathways. The notations are as follows: (↑) in green, metabolite higher in the lorlatinib group than in control group; (↓) in red, metabolite lower in the lorlatinib group than in control group. The related metabolic pathways are graphed in blue boxes.

Four-hundred and ninety-one (491) potential biomarkers were obtained for further analysis by refining the above result based on the additional filtering by the criteria of *p* < 0.05 and fold change (FC) > 2 or < 0.5. The accurate mass charge ratio of all potential biomarkers were entered into a search of the online Human Metabolome database (https://hmdb.ca/) for putative identification of biomarkers. After converting the biomarker from HMDB ID to KEGG ID, 360 biomarkers were enriched in the KEGG pathway and mapped to the metabolic pathways in the metabolomics data analysis platform, MetaboAnalyst 3.0. As shown in Figure 3, we identified 56 biomarkers related to metabolic pathways, of which the most relevant pathways were selected after comprehensive consideration of impact factors and raw_p; these were Sphingolipid metabolism, Glycerophospholipid metabolism, Thiamine metabolism and Synthesis and degradation of ketone bodies. These metabolic pathways hit 9 significant metabolites, namely: Acetoacetyl-CoA (S)-3-Hydroxy-3-methylglutaryl-CoA, Dihydroceramide, Sphingosine, L-Cysteine, Thiamin diphosphate, CDP-ethanolamine, Phosphatidylcholine and Choline, as depicted by the schematic diagram of the metabolic pathways related to lorlatinib (Figure 4).

### Gene Ontology Enrichment Analyses and Kyoto Encyclopedia of Genes and Genomes Pathway Analysis of Differential Genes

**FIGURE 5:**
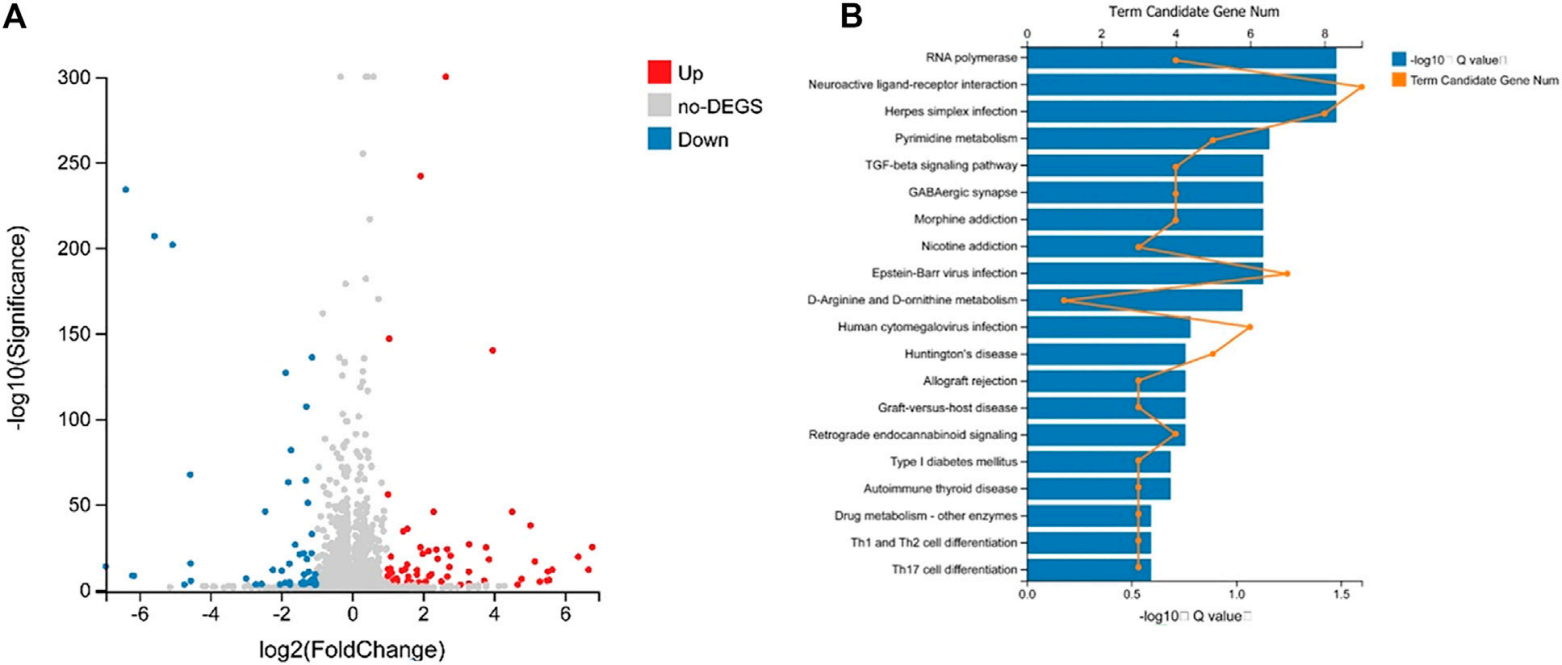
Volcano plot analysis of differently expressed miRNA **(A)** and differential gene KEGG Pathway enrichment histogram **(B)**.

In the preliminary experiment, we sequenced the RNA of the control group and the lorlatinib group mice (Chen et al., 2020). By using DEGseq algorithm, |log2Fold Change| ≥1 and Adjusted *p* value ≤0.001 as the screening criteria, 126 differentially expressed genes were obtained. Among them, there were 70 genes that were significantly up-regulated and 50 genes that were down-regulated (*p* < 0.01). Volcano plots (Figure 5A) were created to quickly identify meaningful changes from within a very large set of genes. According to the GO and KEGG annotation results and the official classifications, we classified the differential genes by function before using the phyper function in the R software package for enrichment analysis. These differentially expressed genes are involved in 23 biological processes, which mainly affect cellular processes, biological regulation, and multicellular organismal processes. The molecular functions of the identified genes mainly involve binding, catalytic activity, and signal transducer activity. The remaining 12 pathways were those of cellular components. Twenty-four pathways were significantly enriched, 20 of which are shown in Figure 5B, these included Neuroactive ligand-receptor interaction, RNA polymerase, Herpes simplex infection, Pyrimidine metabolism and Epstein-Barr virus infection.

### Expression of Key Proteins Within the Blood-Brain Barrier of Mice After Lorlatinib Administration

**FIGURE 6:**
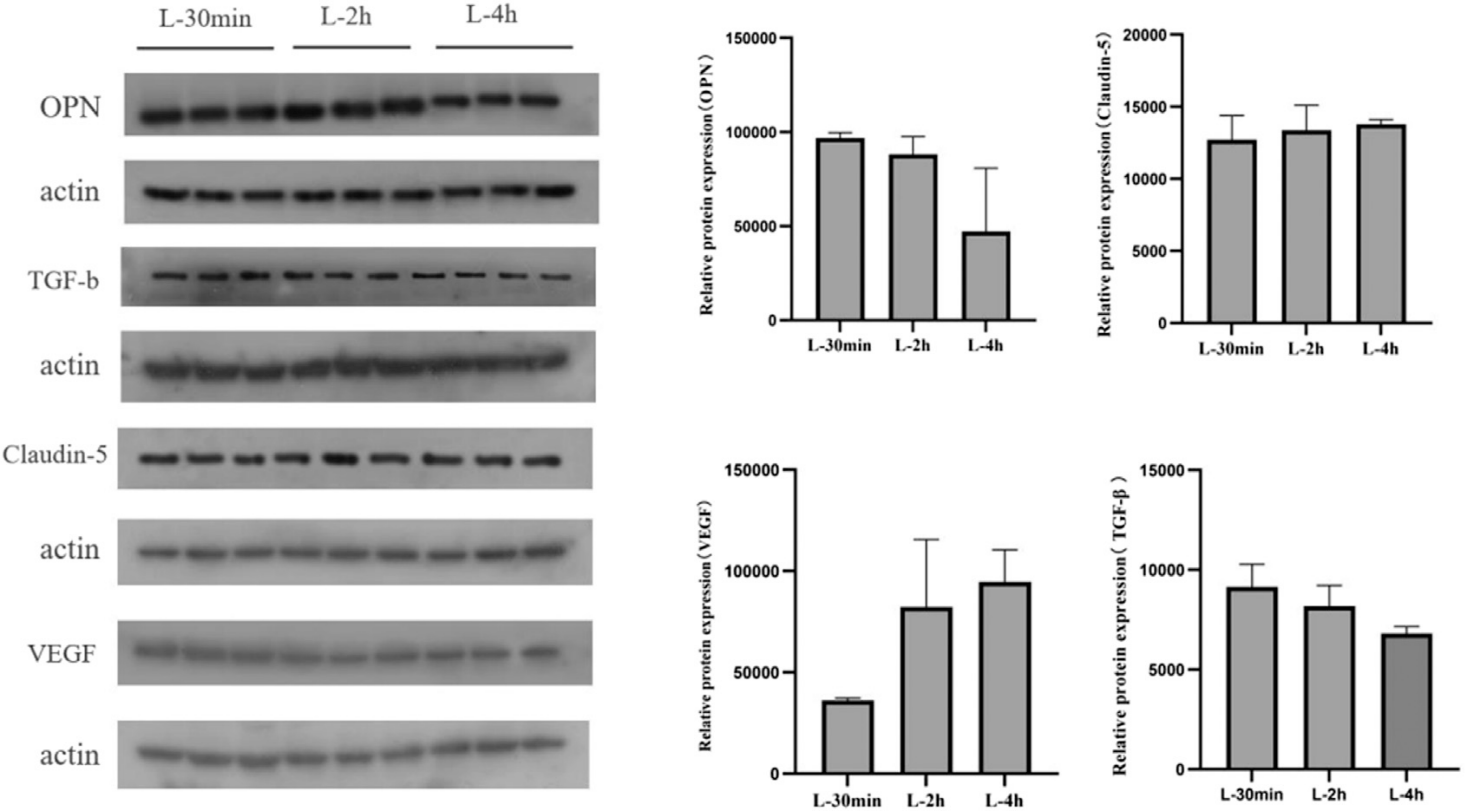
Expression of key proteins in blood-brain barrier after lorlatinib administration.

The expression of OPN protein in the brain tissue of mice gradually decreased with increasing time after administration of lorlatinib. Claudin-5 protein levels did not change significantly within 4 h after lorlatinib administration, and vegf protein was up-regulated within 4 h after administration. Finally, TGF-b was significantly down-regulated after drug administration (Figure 6).

### Metabolite-Reaction-Enzyme-Gene Interaction Network Construction and Analysis

**FIGURE 7:**
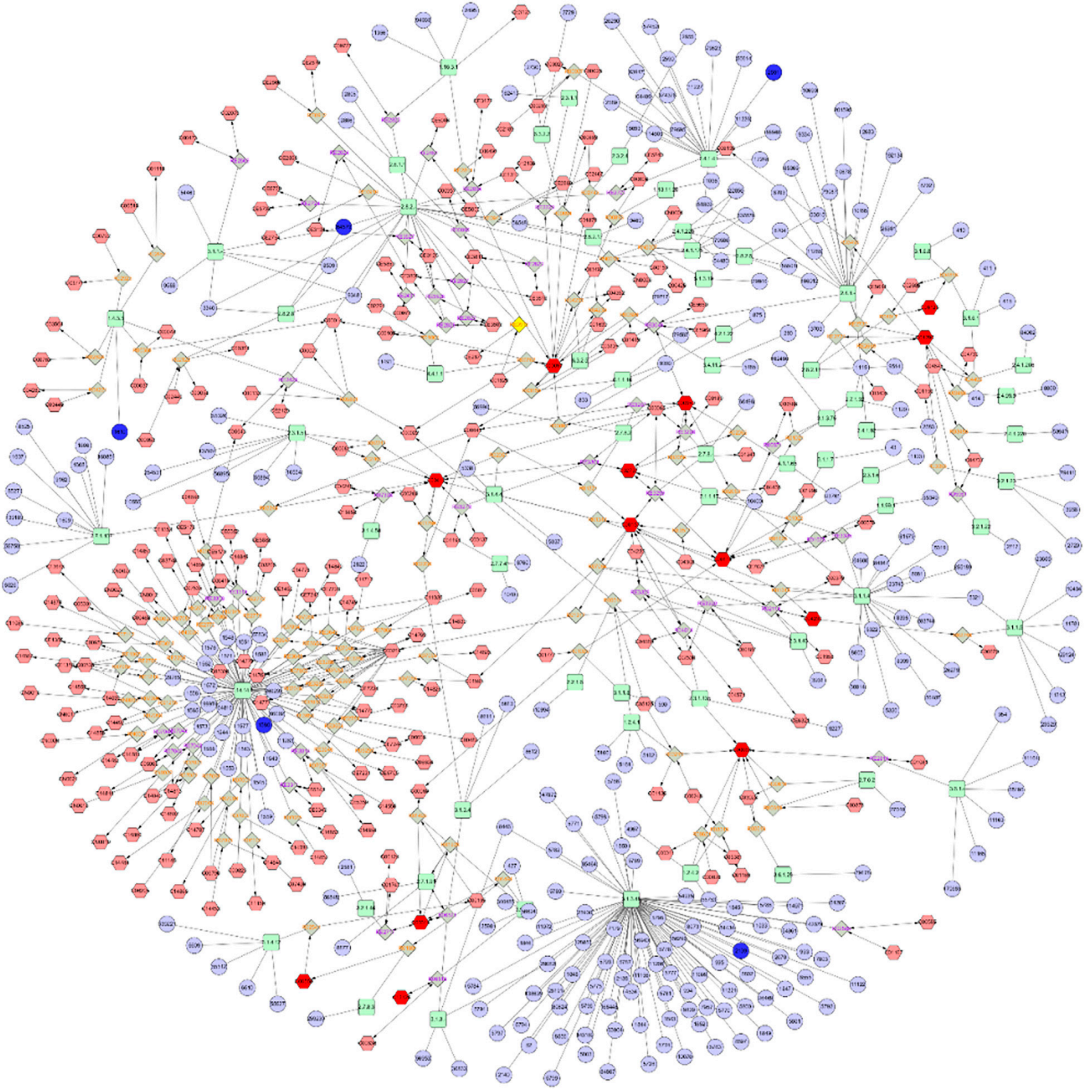
Metabolite-To-Gene interaction network.

Combining metabolomics with transcriptomics, a previously undescribed Metabolite-Reaction-Enzyme-Gene interaction network was constructed by searching for correlations between genetic expression profiles and metabolite accumulation profiles. As shown in Figure 7, the Metabolite-To-Gene interaction network consisted of 13 metabolites which were identified in this study and 5 genes which had been revealed to be important in previous research (Chen et al., 2020). These networks also served to identify and validate a select number of genes and metabolites likely to contribute to combatting drug-resistant tumors and promoting blood-brain barrier permeability.

### Lorlatinib Concentration in Blood and Brain

Mean serum concentration-time curves, upon which the pharmacokinetic parameters and the tissue distribution calculations were based, have been published previously (Chen et al., 2019). The plasma concentration curve shows two-compartment pharmacokinetic characteristics. The ratio of brain lorlatinib concentration to blood concentration in 48 samples was calculated, giving an average of 0.70 (standard deviation of 0.20) and a 90th and 10th percentile of 0.90 and 0.39, respectively. These findings indicated that there was significant individual variation in the distribution of lorlatinib in brain.

### Artificial Neural Network Construction

**FIGURE 8:**
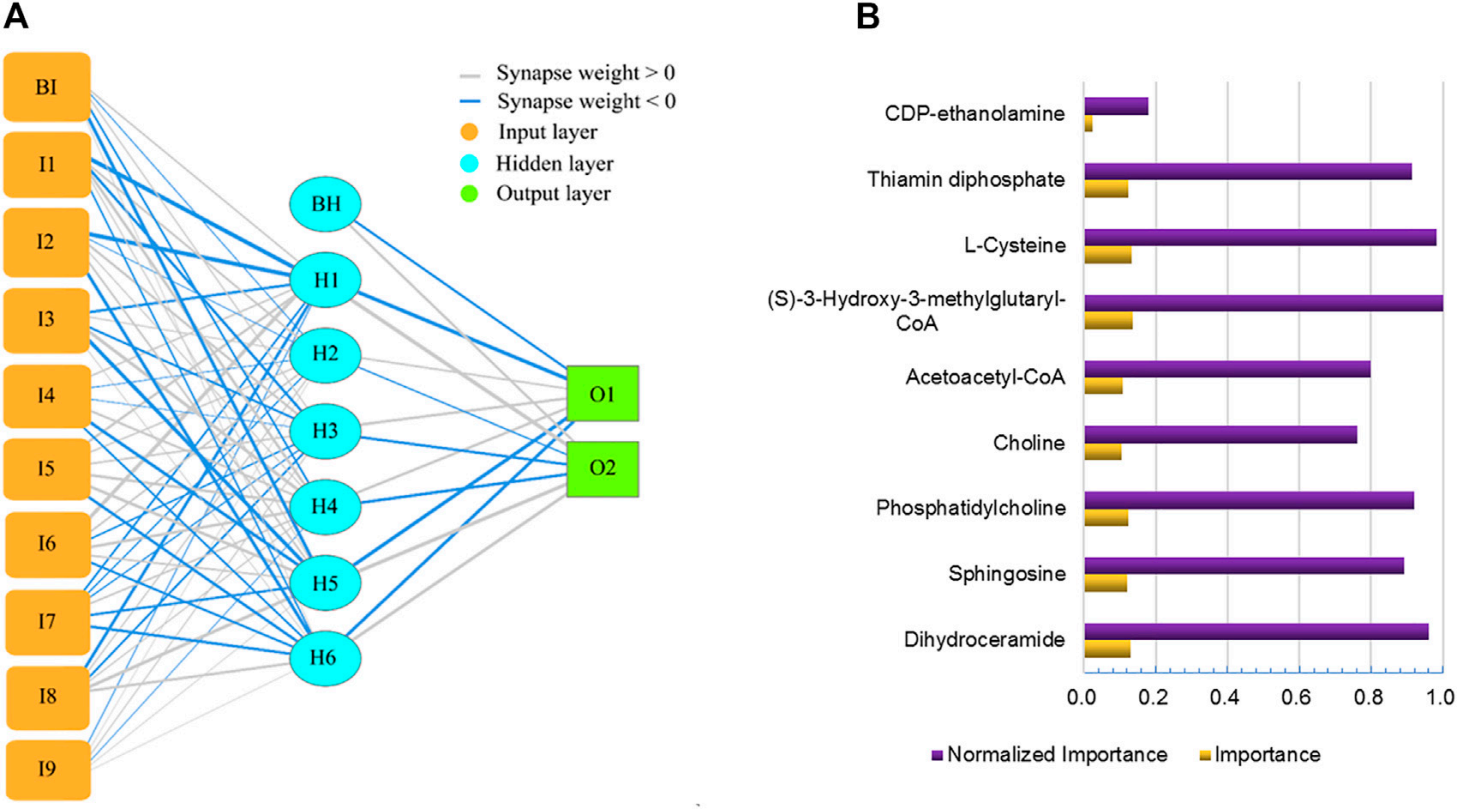
Artificial neural network for predicting brain-blood distribution coefficient **(A)** and the importance of metabolics in the neural network **(B)**.

An artificial neural network (Figure 8A) was created with 9 inputs, one hidden layer, and one output layer. The hidden layer had 6 nodes. The output layer had 2 nodes since we needed to implement a binary classification of the blood-brain distribution coefficient, where there could only be a high-coefficient level or low-coefficient level. The hyperbolic tangent function, a nonlinear activation function that outputs values between −1.0 and 1.0, was used for connection between the input layer and the hidden layer. The sigmoid function, which can transform the range of combined inputs to a range between 0 and 1, was used as the Output layer activation function. This neural network architecture is more suitable for the nonlinear boundaries formed by complex metabolic processes. The classification table (Table 1) shows the practical results of using the neural network. In Figure 8B, we provide the importance of independent metabolic biomarkers as different measures of the extent to which the network’s model-predicted classification of brain-blood distribution coefficient is altered for different values of the independent metabolic biomarker. Normalized importance is simply the importance value divided by the importance values of (S)-3-Hydroxy-3-methylglutaryl-CoA, which had the largest importance value among all metabolic biomarker variables.

**TABLE 1:** The classification table of the practical results.

Sample	Observed	Predicted		Percent correct (%)
		0	1	
Training	0	14	2	87.5
	1	2	15	88.2
	Overall Percent	48.5%	51.5%	87.9
Holdout	0	6	1	85.7
	1	0	7	100.0
	Overall Percent	42.9%	57.1%	92.9

## Discussion

Using structure-based drug design, along with the overcoming of synthetic challenges, the highly potent macrocyclic ALK inhibitor, lorlatinib, was discovered. Lorlatinib is characterized by a high degree of kinase selectivity, good passive permeability and a low propensity for p-glycoprotein 1-mediated efflux (Johnson et al., 2014). The above characteristics have been further confirmed in clinical trials: lorlatinib had a mean cerebrospinal fluid to plasma concentration ratio of 0.75 confirming significant CNS penetration, had an IC response rate of 63% in brain metastasis patients previously administered with at least one ALK inhibitor, confirming superior CNS activity compared to first-generation TKIs (Serritella and Bestvina, 2020; Xia et al., 2020).

To further clarify the explicit effect and underlying mechanism of lorlatinib, especially regarding its intracranial activity, metabolomic profiles were investigated and combined with previous transcriptomics research (Chen et al., 2020), rendering a panoramic view of the interaction between lorlatinib and the body. In this research project, 9 noteworthy differential metabolites contributing to the altered metabolic profiles of experimental groups were identified, and they were enriched in 4 major metabolic pathways, namely, Sphingolipid metabolism, Glycerophospholipid metabolism, Thiamine metabolism and Synthesis and degradation of ketone bodies.

Several groups of lipids, such as sphingosines (Yanagida et al., 2017), alkylglucosides, oxidized lipids and ether lipids have been identified as non-toxic and reversible tight junction (TJ) modulators (Johnson et al., 2008). Lorlatinib is linked closely with regulating sphingolipid, which has a notable role in membrane integrity, vasculogenesis, and immune cell infiltration into the brain (Gu et al., 2020).

Ceramide, the precursor of all sphingolipids and the central molecule of sphingolipid metabolism, can be synthesized by four different pathways involving reactions during which DES introduces a double bond to the dihydroceramide molecule. Sphingosine is directly phosphorylated by sphingosine kinases (SphK1 and SphK2) to generate sphingosine-1-phosphate (S1P) (Gomez-Muñoz et al., 2016). It is worth noting that the role of SphK1 and S1P was confirmed to be critical in the maintenance of endothelial barriers. Sphingosine kinase-1 modulates vascular endothelial permeability at the surface of the blood brain barrier (BBB) (Gu et al., 2020). S1P, produced by SphK1 catalysis, has been shown to bring a rapid and drastic reduction in the focal adhesion strength and barrier tightness of brain endothelial cells (Wiltshire et al., 2016). In the comparison between the lorlatinib group and the control group in the present study, sphingosine levels in the lorlatinib group decreased significantly, while dihydroceramide increased considerably. These data led us to infer that the remarkable lorlatinib characteristic of good intracranial activity was contributed to by regulation of S1P in sphingolipid metabolism. While sphingosine and S1P can mutually transform, lorlatinib blocks the conversion of S1P to sphingosine, which in this case has manifested as a decrease in sphingosine levels. The described phenomenon is very likely to be accompanied by an increase in S1P levels, thereby rapidly and acutely reducing endothelial barrier resistance and enhancing the intracranial activity of lorlatinib. Considering the degree of correlation between the above two compounds and BBp.

P-glycoprotein, an ATP-binding cassette (ABC) transporter, which is a major pump that transports promiscuous xenobiotics out of cells, associates with multidrug resistance (MDR) due to overexpression (Cannon et al., 2012; Mollazadeh et al., 2018; Ren and Gray, 2019). In a previous study, through RNA sequencing, we confirmed that lorlatinib did not exhibit a significant regulatory effect on the p-glycoprotein via mRNA transcription (Chen et al., 2020). However, sphingolipid, signaling via S1P and acting via S1PR1, appears to induce a fast and reversible regulatory effect resulting in low p-glycoprotein pump activity level and an improvement in the delivery of small-molecule compounds to the brain (Cannon et al., 2012). Sphingolipids are signaling molecules involved in inflammatory responses (Mesev et al., 2017). A S1P analogue could alter BBB efflux transport by inhibiting the S1P receptor 1-mediated inflammation and alleviating P-gp overexpression in rat hippocampus (Gao et al., 2018). In the present study, the enrichment of sphingolipid metabolism pathways suggested that lorlatinib inhibited the function of P-glycoprotein, which could be one of the reasons why lorlatinib is still effective in ceritinib-resistant patients with P-gp over-expression (Katayama et al., 2016). In combination medication therapies, it was also possible that lorlatinib had a strong reversal effect on multidrug resistance, because P-gp efflux of drugs is the main cause of multidrug resistance. However, it has been identified that P-glycoprotein/ABCB1 in the BBB remained the major obstacle to brain accumulation of lorlatinib (Li et al., 2018); simultaneous administration of P-gp inhibitors could greatly boost absolute brain levels of lorlatinib (Li et al., 2019a).

Tight junctions play a crucial role in regulating blood-brain barrier permeability. The main modulators acting directly on tight junction components include occludin (Yuan et al., 2020), claudin-5 (Greene et al., 2019), zonulin and E-cadherin (Deli Mária, 2009; Hashimoto and Campbell, 2020), the expression levels of which are closely related to cerebral microvascular permeability. In preliminary studies, we used a PCR method to confirm that SPP1, VEGF, TGF-β and claudin are down-regulated 1 day and 7 days after lorlatinib administration. In order to present the correlation of lorlatinib with tight junction proteins in a panoramic view, in this study, western blotting was applied to explore the changes in tight junction protein levels within the first few hours after administration.

The results demonstrated that levels of OPN and TGF-β had a gradual downward trend within 30 min to 4 h after lorlatinib dosing, whereas VEGF had a clear upward trend, and no significant changes were shown in Claudin-5 levels. Only OPN and TGF-β levels decreased within a short time after lorlatinib administration, indicating that OPN and TGF-β are directly and potently affected by lorlatinib. OPN plays an essential role in tight junctions by affecting occluding via a well-defined pathway (Woo et al., 2019). There are also elusive underlying mechanisms regarding OPN’s regulation of ZO-1, claudin-5 (Zhang et al., 2018) and of TGF‐βmodulating claudin (Wang et al., 2020). The variation in response of claudin-5 at different time periods is probably due to the influence of requiring multiple signal pathway transmissions, which maybe also be the major reason for a feedback increase of VEGF at the initial time period after lorlatinib administration.

To obtain a more comprehensive understanding of the regulatory mechanisms of lorlatinib, a Gene-To-Metabolite interaction network (Figure 7) was constructed through Cytoscape. The complex network contained five genes, which were CYP4B1, GALNT3, DAO, NDST4, EYA2, and 13 metabolites, which were Sphingomyelin, Dihydroceramide, Sphingosine, Thiamin diphosphate, 1-Acyl-sn-glycero-3-phosphocholine, Phosphatidylcholine, Choline, Phosphatidate, Phosphatidylserine, Phosphatidylethanolamine, L-Cysteine, beta-D-Galactosyl-1,4-beta-D-Glucosylceramide and Sulfatide. Related genes encode enzymes belonging to different superfamilies, catalyzing many reactions involved in: metabolism of certain xenobiotics (Lim et al., 2020; Baer and Rettie, 2006), posttranslational modification of protein (Takashi and Fukumoto, 2020), N-methyl-d-aspartate receptor regulation, glutamate metabolism (Yang et al., 2013), modification in the heparan sulfate biosynthetic pathway (Li et al., 2018) and transcriptional activation (Devi Maharjan et al., 2019). The results of the presented integrated metabolomics and transcriptomics analysis prove that the pathway is concentrated on Sphingolipid metabolism and Glycerophospholipid metabolism, which is consistent with the enrichment results. In addition to the four highly enriched pathways described in item 3.1, the differential metabolites in the Gene-To-Metabolite interaction network also involve multiple pathways such as Metabolism of xenobiotics by cytochrome P450, D-Arginine and D-ornithine metabolism, Arachidonic acid metabolism, and Glycine, serine and threonine metabolism. A variety of substances related to nodes in the Gene-To-Metabolite interaction network such as Eyes Absents (EYA) (Tadjuidje et al., 2012), polypeptide N-acetylgalactosaminyl transferase 3 (GalNAc-T3) (Guo et al., 2016), amino acids and fatty acid oxidation (Li et al., 2019b) and phosphatidylcholine hydroperoxide (Nakagawa et al., 2011) were all essential requirements for or regulators of endothelial cells, suggesting their inextricable linkage to the permeability of the blood-brain barrier. The network pharmacology results indicated that lorlatinib could hit multiple targets in multiple ways, which lead more brain distribution and higher intracranial effectiveness.

## Conclusion

The percentage scores of correct predictions in training and testing of the artificial neural network were both over 85%, which indicate that the deep learning provides an effective pathway by which to solve the nonlinear problem of prediction. At the same time, it also exhibits that the metabolic biomarkers screened play a key role in predicting the brain-blood distribution coefficient of lorlatinib and revealing the concentration of the drug in the brain. The identification of markers and models also provide a reference for lorlatinib to treat tumor brain metastases in the clinic. However, due to the limited number of samples, this model requires further verification on a large scale.

## Data Availability

The data supporting the conclusions of this article will be made available by the authors, upon request, without undue reservation.

## Glossary

NSCLC: non-small-cell lung cancer
ALK: anaplastic lymphoma kinase
UPLC-Q/TOF-MS: ultra-performance liquid chromatography and quadrupole/time-of-flight mass spectrometry
QC: quality control
ESI: electrospray ionization
PCA: Principal Component Analysis
OPLS-DA: Orthogonal Partial Least Squares Discriminant Analysis
FC: fold change
GO: Gene ontology
KEGG: Kyoto Encyclopedia of Genes and Genomes
TJ: tight junction
S1P: sphingosine-1-phosphate
BBB: blood brain barrier
ABC: ATP-binding cassette
MDR: multidrug resistance
EYA: Eyes Absents
